# Impact of surgical management in cases of intraoperative membrane perforation during a sinus lift procedure: a follow-up on bone graft stability and implant success

**DOI:** 10.1186/s40729-018-0116-8

**Published:** 2018-02-05

**Authors:** Benedicta E. Beck-Broichsitter, Dorothea Westhoff, Eleonore Behrens, Jörg Wiltfang, Stephan T. Becker

**Affiliations:** 1 Department of Oral and Maxillofacial Surgery, Charité–University Medical Center Berlin, Augustenburger Platz 1, 13353 Berlin, Germany; 2 Department of Oral and Maxillofacial Surgery, Schleswig-Holstein University Hospital, Arnold-Heller-Straße 3, Haus 26, 24105 Kiel, Germany

**Keywords:** Sinus floor elevation, Intraoperative complication, Perforation, Schneiderian membrane, Implant survival

## Abstract

**Background:**

Until now, sinus floor elevation represents the gold standard procedure in the atrophic maxilla in order to facilitate dental implant insertion. Although the procedure remains highly predictive, the perforation of the Schneiderian membrane might compromise the stability of the augmented bone and implant success due to chronic sinus infection. The aim of this retrospective cohort study was to show that a membrane tear, if detected and surgically properly addressed, has no influence on the survival of dental implants and bone resorption in the augmented area.

**Methods:**

Thirty-one patients with 39 perforations could be included in this evaluation, and a control group of 32 patients with 40 sinus lift procedures without complications were compared regarding the radiographically determined development of bone level, peri-implant infection, and implant loss.

**Results:**

Implant survival was 98.9% in the perforation group over an observation period of 2.7 (± 2.03) years compared to 100% in the control group after 1.8 (± 1.57) years. The residual bone level was significantly lower in the perforation group (*p* = 0.05) but showed no difference direct postoperatively (*p* = 0.7851) or in the follow-up assessment (*p* = 0.2338). Bone resorption remained not different between both groups (*p* = 0.945). A two-stage procedure was more frequent in the perforation group (*p* = 0.0003) as well as peri-implantitis (*p* = 0.0004).

**Conclusions:**

Within the limits of our study, the perforation of the Schneiderian membrane did not have a negative impact on long-term graft stability or the overall implant survival.

## Background

Sinus floor elevation procedures have become a predictable and successful treatment, performed when the maxillary alveolar ridge is atrophied and the bone height is not sufficient for primary implantation. If the postoperative course remains uneventful, the outcome is highly predictable [1–3]. However, complications may have a negative impact on the overall treatment success. As a common complication, perforation of the Schneiderian membrane occurs in 12 to 44% of cases depending on the literature [2, 4–6], with an average of 20 to 25% [7–9] in all cases due to septa morphological aspects of the membrane or other pathologic conditions; the perforation itself represents the major intraoperative complication despite common complications, such as postoperative infection [5, 10].

Still, it is not completely clear to what extent these complications influence implant survival or might impact the augmented material in the sinus. To evaluate the impact of early-onset complications during implant insertion on the implant success, Becker et al. published a follow-up study evaluating the first year after a sinus lift procedure [11], which did not reveal a negative impact on implant survival after an observation period of 162 days. In contrast to these results, a study by Nolan et al. retrospectively re-assessed a total of 359 sinus augmentation procedures with a perforation rate of 41.8% (150 patients) at least 1 year after implant loading and reported a graft failure rate of 6.7%, in which 70.8% of membranes were perforated. In a study by Sakkas et al. [12], membrane tear (perforation rate 10.8%) was slightly not significantly associated with postoperative complications in 105 sinus lift procedures. A highly significant connection was shown in a study by Schwartz-Arad et al. [5], but these complications reportedly had no impact on implant survival.

In this retrospective study, the patient cohort with perforations of the Schneiderian membrane from the previously reported study [11] was re-evaluated to specifically assess local bone remodeling and resorption processes in the augmented area, signs of chronic infection of the sinus, and implant survival compared to a group of patients without a membrane tear over a longer time period. We hypothesize that if detected and properly handled surgically or with an adequate adaption of the surgical protocol, a perforation of the Schneiderian membrane would not endanger the outcome parameters of stable bone augmentation and promotion of signs of peri-implant disease and implant loss.

## Methods

### Patient recruitment

In accordance with the WMA Declaration of Helsinki—Ethical Principles for Medical Research Involving Human Subjects, approval was given by the local ethics committee of the Christian-Albrechts-University in Kiel (AZ 132/10). All patients gave informed written consent to participate.

A total of 201 sinus floor elevation procedures, which were performed from 2005 to 2006 in the Department of Oral and Maxillofacial Surgery of the University Hospital of Kiel, were primarily included in this retrospective cohort study. Within this cohort, 41 perforations (20.4%) of the Schneiderian membrane in 33 patients (21 female, 12 male) occurred. One patient was deceased, and one patient did not engage in the follow-up offered by the department. After exclusion of these two patients, a total of 31 patients aged 60.86 (± 11.21) years with 39 perforations were available to participate in regular recall examinations with an average observation time of 2.69 years (± 2.04 years). According to this study group, 32 patients with 40 sinus lift procedures without perforations aged 58.76 years (± 9.43) were randomized from the cohort to represent the control group (average observation time 2.14 years ± 1.85 years). Patients were recruited from the established recall system in the department. Requirements of inclusion were engagement in at least three follow-up visits after dental implantation and receiving dental implants in the department, if a two-stage procedure was performed. The inclusion rate of patients in the control group was 33.28%. In total, 56.16% of patients (54 patients) within the control group had not completed the follow-up visits for various reasons (relocation, impairment because of age, follow-up performed elsewhere) and therefore were excluded. Sixteen patients received implants in other private practices (16.64%), and 2 patients were deceased.

### Medical record assessment

The manufacturer and position of implants were previously extracted from surgical reports in the medical record, as were in-house treated implant failures and consecutive explanation procedures. Vertical bone augmentation was additionally classified dependent on donor site (none/linea obliqua/iliac crest/scapula).

### Implant therapy

Three different oral and maxillofacial surgeons performed the sinus lift procedure with an external approach according to comparable surgical standards and inserted all implants examined in this study in a submerged protocol with uncovering after 3–4 months due to the manufacturer’s surgical recommendations. Specifically, a total of 35 external sinus floor elevations were performed through a bone window in the facial aspect of the maxillary sinus. The internal sinus lift approach was applied once. In four patients, sinus floor elevation was accompanied with a LeFort I osteotomy or in one case with a reconstruction of the maxilla after tumor surgery. Preexisting defects were assumed due to trauma or previous surgical interventions in three operation sites.

For sinus floor augmentation including defects of less than 2 cm^3^, bone filter material and bone substitute [13, 14] were applied. If the defect exceeded 2 cm^3^, only autologous bone was used.

Perforations up to a diameter of 5 mm in size were covered with a BioGide membrane (Geistlich, Wolhusen, Switzerland), and perforations beyond a diameter of 5 mm up to 10 mm were additionally stitched with resorbable sutures (Vicryl 6-0, Ethicon, Norderstedt, Germany) while larger defects led to termination of the procedure. Only in exceptional cases were perforations left untreated or sealed with fibrin glue.

Implants were either placed in a one-stage procedure accompanied with the sinus floor elevation or in a two-stage procedure if primary stability might not be achieved due to the bone being present.

Patients received antibacterial mouth rinse, systemic antibiotics, nose drops, and inhalants from 7 to 10 days beginning directly after the operation. Sutures were removed 7 to 10 days after the surgical procedure. All patients were instructed how to maintain appropriate oral hygiene directly after surgical intervention and were re-instructed after the uncovering procedure and during recall sessions. Patients were further asked to join for regular recall examinations after prosthodontic rehabilitation and thereafter each year. Six months after sinus floor elevation, panoramic radiographs were made.

### Clinical assessment

One independent oral and maxillofacial surgeon performed the clinical follow-up examinations according to a standardized protocol. A peri-implant probing including probing pocket depths and recessions on four sites of each implant was assessed as was bleeding on probing (BOP) to determine the status of oral hygiene objectively. Signs of gingivitis and pus suppuration were also recorded. The criteria of peri-implantitis were based on those published by Ong et al. [15]: peri-implant probing depth ≥ 5 mm and bleeding on probing and/or suppuration and radiographic bone loss ≥ 2.5 mm.

**Fig. 1:**
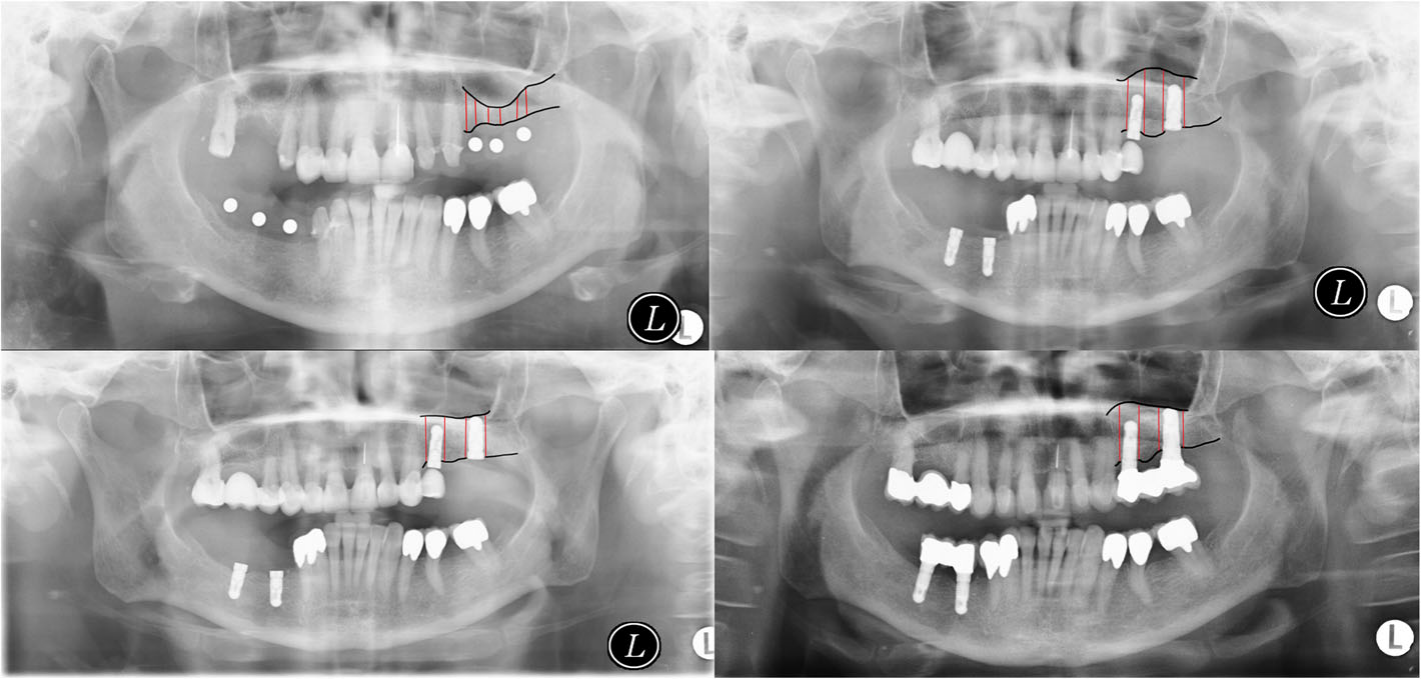
Bone levels after sinus floor elevation

Based on panoramic radiographs, marginal bone levels were measured on the distal and mesial sites of each implant. Bone loss was calculated based on the known implant length and the radiographic magnification factors accordingly. Distances were measured to the nearest millimeter. Bone levels after sinus floor elevation were compared to bone levels in follow-up (Fig. 1).

The implant success rate was defined as the absence of patients’ complaints and objective signs of peri-implant inflammation (bleeding on probing, peri-implantitis, dehiscence defects, and implant stability).

### Statistical assessment

Statistical data analyses were performed using GraphPad Prism version 6.0 (GraphPad Software, La Jolla, CA, USA). Descriptive statistics (mean value, standard deviation, and percentage distribution) were calculated, and the data were checked for Gaussian distribution applying the Shapiro-Wilk test. Comparisons between the groups with and without perforation were assessed with non-parametric statistic testing (Mann-Whitney-*U*-Wilcoxon). Fisher’s exact test was applied for combinations of factors, and implant survival was displayed in a Kaplan-Meier plot. Multiple comparisons according to Tukey were applied in cases of further subdivision of the datasets. If the probability of error was less than 5%, the result was presented as statistically significant.

## Results

### Descriptive statistical evaluation

#### Perforation group

The mean control interval was 2.69 (± 2.03) years. At the time of the follow-up examination, the average age was 59.95 (± 11.82) years.

**Fig. 2:**
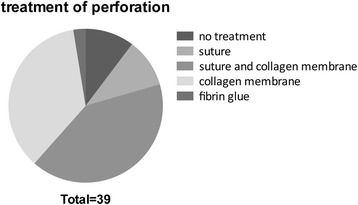
Overview of the perforation treatment in the study group

**Fig. 3:**
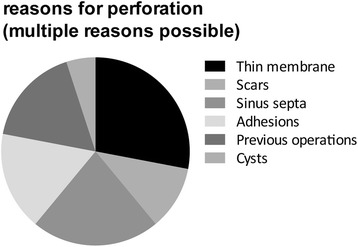
Reasons for perforations

In the remaining collective of 31 patients (96.97%; 12 males (37.54%) and 19 females (59.43%)), a total of 92 implants were inserted. The overview of perforation treatment in the study group is given in Fig. 2, and Fig. 3 depicts the reasons for perforations. Eleven implants (11.96%) were inserted in a one-stage procedure, whereas the remaining 81 implants (88.04%) were inserted in a second surgical intervention. One implant (1.09%) had to be removed due to wound infection 3 months after implant insertion. After a 6-month healing period, the implant could be successfully replaced.

Many implants were purchased from Nobel Biocare with a total of 33 (35.87%), followed by Camlog with 24 (26.09%), Straumann with 18 (19.57%), Ankylos with 10 (10.87%), and Frialit with 5 implants (5.43%). The Astra implant system was applied twice (2.17%).

Twelve implants showed a probing depth above 3 mm; bleeding on probing was positive in 7 implants, and 6 implants showed signs of peri-implantitis or patients had previously had peri-implant surgery.

#### Control group

The patients’ (16 female, 15 male) mean age included in this group was 59.32 years (± 11.34 years) with a mean observation period of 1.80 years (± 1.57 years).

A total of 83 implants were inserted, of which 30 implants were inserted in a one-stage procedure and 53 in a two-stage procedure. No implant had to be removed during the observation period.

Forty-two implants were purchased from Camlog (50.6%), followed by Nobel Biocare with 22 implants (26.51%), Straumann with 18 implants (21.69%), and one Astra implant (1.2%). No signs of inflammation or peri-implantitis were detected in the clinical examinations.

### Group comparison

The preferred implant positions in both groups are depicted in Table 1, showing a homogenous distribution when comparing both groups. Table 2 provides an overview of bone graft origin. In the control group, the majority of procedures (65.1%) did not require an additional bone graft, whereas 59.8% of surgical interventions required iliac crest in the perforation group.

**Table 1:** Distribution of implant positions

Implant position	3		4		5		6		7		8	
Perforation group	7	7.6%	23	25.0%	27	29.4%	30	32.6%	5	5.4%	0	0%
Control group	9	10.8%	20	24.1%	25	30.1%	21	25.3%	7	8.4%	1	1.2%

**Table 2:** Origin of bone graft

Origin of bone graft	No bone graft		Linea obliqua		Iliac crest		Scapula flap	
Perforation group	15	16.3%	22	23.9%	55	59.8%	0	0%
Control group	54	65.1%	11	13.3%	13	15.7%	5	6.02%

**Fig. 4:**
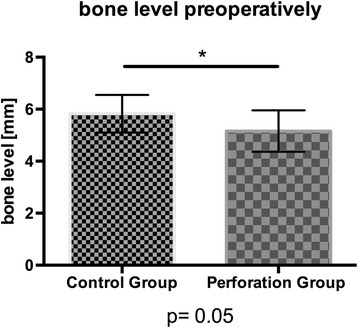
The initial bone level of the control group and the perforation group

**Fig. 5:**
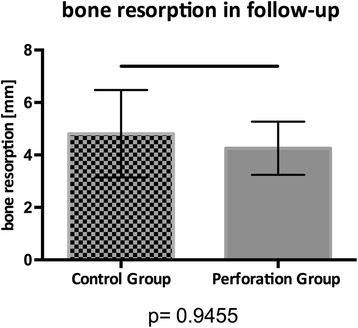
Bone resorption in the follow-up of the control group and the perforation group

The initial bone level differed significantly (*p* = 0.05) between both groups with a median value of 5.69 mm in the study group and 3.87 mm in the control group (Fig. 4). A Mann-Whitney-*U*-Wilcoxon test revealed no significant difference between bone level postoperatively (*p* = 0.7851; median value control group 17.40 mm; median value perforation group 16.91 mm), in follow-up (*p* = 0.2238; median value control group 13.88 mm; median value perforation group 13.31 mm), or for bone resorption (*p* = 0.9455, median value study/perforation group 3.45 mm; Fig. 5). The data are further summarized in Table 3.

**Table 3:** Data summary of bone level development

	Bone level preoperatively		Bone level postoperatively		Bone level follow-up		Bone resorption	
	Perforation group	Control group	Perforation group	Control group	Perforation group	Control group	Perforation group	Control group
Median value	3.87	5.69	16.91	17.40	13.31	13.88	3.45	3.45
Standard deviation	3.79	3.32	5.06	6.04	2.39	4.02	4.84	7.64
*p* value	0.05		0.7851		0.2235		0.9455	

Groups were further subdivided and separated at an initial bone level of 4 mm, above which the sinus lift procedure is considered a relative indication for achievement of primary stability [16]. Regarding the height of augmented sinus floor postoperatively, a multiple comparison analysis revealed a significant difference between the groups. The adjusted *p* values of multiple comparisons are depicted in Table 4. Interestingly, only two group comparisons revealed a relevant difference. Multiple comparisons of bone resorption in subgroups did not reveal a statistically significant difference (*p* = 0.1418).

**Table 4:** Multiple comparisons of subgroups: postoperative bone level

Adjusted *p* values multiple comparison	Control group bone level < 4 mm		
Control group bone level > 4 mm	0.0453	Control group bone level > 4 mm	
Perforation group bone level < 4 mm	0.3174	0.7586	Study group bone level < 4 mm
Perforation group bone level > 4 mm	0.0203	0.9679	0.5144

Fisher’s exact test revealed a statistically significant difference of proportions of one-stage versus two-stage procedures due to the presence of perforation (*p* = 0.0003; Table 5) and a significantly higher trend for peri-implantitis in patients with perforation (*p* = 0.0004; Table 6).

**Table 5:** Fisher’s exact test: surgical strategy dependent on membrane perforation (*p* = 0.0003)

	One-stage procedure	Two-stage procedure
Perforation group	11	81
Control group	30	53

**Table 6:** Fisher’s exact test: incidence of peri-implant disease after sinus lifting procedure with and without membrane perforation (*p* = 0.0004)

	Peri-implantitis	No peri-implantitis
Perforation group	12	80
Control group	0	83

## Discussion

The aim of this retrospective cohort study was to evaluate the impact of intraoperative perforations of the Schneiderian membrane during sinus floor elevation on the stability of the augmented area and its influence on osseointegration after implant insertion. Therefore, we could re-assess a patient cohort of originally 34 patients with 41 perforations and compare their outcome with a control group of patients with sinus floor elevation but without membrane tear, offering a long-term perspective over a range of 1.8 years in the control group to 2.7 years in the study group.

Although the height of the alveolar crest differed significantly, the total postoperative height of augmented and residual bone was on a comparable level allowing for sufficient elevation of the sinus floor to insert dental implants independently of a perforation. This fact remains reassuring as the latter represents the mostly common complication with a reported variation of 10 to 44% [5, 6, 8, 12, 17–23].

In this context, the surgical experience and the tissue quality, e.g., scarring due to previous procedures or local inflammation, might lead to a high probability of Schneiderian membrane perforation [16, 22, 24, 25] with anatomical variations, such as sinus septa or thin, vulnerable membrane textures [4, 16, 22, 24, 25]. Some studies have suggested contraindication of sinus floor elevation in patients with anatomical variations such as septa or mucosal swelling [8]. As our results did not indicate any negative impact of membrane tear on the augmented sinus, the results of Nolan et al., including a total of 359 sinus floor augmentations, indicated that graft failure occurred in 6.7% of all procedures and was significantly correlated (*p* = 0.0028) with membrane perforation. Compared to our evaluation, where procedures were, without exception, performed by attending physicians, the perforation rate was twice as high (21 vs. 41%). Surgical experience was ruled out as an influencing factor, as membrane perforations were equally distributed in cases treated by attending physicians compared to those performed by residents [6]. Another reason for the different findings might be due to the differences between the numbers of compared membrane tears (39 perforations vs. 150 perforations), as both evaluations were performed retrospectively.

One implant was lost in the perforation group due to early-onset peri-implantitis, whereas all implants in the control group were still in place. As we had previously prospectively reported on the first 6 months after dental implantation in this cohort [11], there was no further impact of membrane perforation on implant loss for at least 12 to 24 months in this retrospective evaluation. The appearance of peri-implantitis was more often observed in patients who experienced a procedure with an intraoperative perforation. However, this finding might be because the control group did not fully represent the patients without membrane perforation as only one third engaged in a sufficient follow-up. The reduced time span of the patients’ observation time in the control group might be due to the lack of postoperative complications in this group who did have follow-up examinations in the department. Therefore, we cannot conclude whether there is or is not an impact based on the results in our study cohort. Sakkas et al. reported no impact of membrane perforation on the overall implant within a 1-year evaluation [12], whereas Proussaefs et al. reported a decreased implant survival in two-stage procedures and membrane tear compared to intact membranes (69.56 vs. 100%) [10], as did a study published by Khoury [7].

In our study, bone resorption in the augmented area did not differ significantly between sinus lifting procedures with or without perforations. It is now widely accepted that following initial bone remodeling after an augmentation procedure followed by a dental implantation, bone loss within the following 3 years with implants in the interproximal space should be less than 0.5 mm in radiographic evaluations [26, 27]. Consistent with the results of Sakkas et al., there was no impact of bone graft origin or postoperative complications in patients with perforation of the Schneiderian membrane [12], similar to a study by Moreno Vazquez et al., which also did not find a correlation between complications, graft failure, and membrane perforation, assessing 8 years postoperatively [24]. In contrast to these studies, Proussaefs et al. reported a significant negative impact of membrane tear on bone formation in the sinus, more soft tissue formation, and less contact of graft particles to the residual bone [10].

The surgical management in cases of a membrane perforation might also influence the overall postoperative outcome and complications. Although the sinus lifting procedure has been established for many years now, there are no evidence-based guidelines for perforation closure or indications to interrupt the procedure. To date, most existing studies recommend sealing smaller sizes of perforations with membranes (collagen, demineralized laminar bone) or fibrin glue. Additional resorbable sutures in cases of larger perforations are advisable if a complete closure of the perforation is feasible [5, 8, 28] but have not been shown to be superior as the coverage of larger perforations with membranes alone were shown to be effective [9, 12, 28, 29]. A lateral approach in sinus lifting might be obligatory to securely detect and therefore treat a perforation. In the primary assessment of the study, four procedures had to be terminated due to an extensive perforation, thin mucosa, or a retention cyst. After waiting 6 months, the procedure was repeated without any complications [11]. Other studies also recommend interrupting the procedure, when the repair does not seem to be sufficiently possible [7, 17].

In this study, a one-stage procedure was significantly less likely to result in membrane perforation. Implant insertion was immediately performed only if the estimated residual bone quality ensured high primary stability, which was consistent with a study by Cha et al. [3]. Residual bone height between 1 to 3 mm was not favorable for immediate implant insertion after sinus floor elevation with a lateral approach [16]. Therefore, the surgeon should be aware that a two-stage approach includes the risk of further complications relating to the surgical procedure itself. A recently published study revealed a significantly higher risk for soft-tissue complications in cases of a second procedure [30].

Due to the retrospective nature of this study, the management in cases of perforation did not follow a standardized protocol. Most of the studies regarding the outcome after membrane tear rely on retrospectively acquired data, and similar to in our study, with an inhomogeneous study cohort with different approaches in cases of perforation, there were different augmentation procedures, including grafts and grafting material, one- and two-stage procedures and different types of dental implants. Based on our data and regarding the limitations within the design of this study, we might conclude that a perforation of the Schneiderian membrane, if recognized and properly addressed, does not necessarily endanger or negatively impact the stability of the augmented bone or implant survival. To systematically assess the impact of membrane perforation on the augmented sinus and implant survival, prospective studies and higher case numbers should be considered in the future.

## Conclusions

In conclusion, and within the limits of its retrospective nature, our study implies that in cases of intraoperative perforation of the Schneiderian membrane, a consequent surgical assessment and treatment might avoid complications regarding graft stability and implant survival. Two-stage procedures might be appropriate if primary stability does not seem to be achievable. Augmentation of the sinus floor might be possible even in cases of perforation. A negative impact on the bone graft itself or on remodeling processes in follow-up could not have been shown, but prospective long-term studies should be performed to deliver reliable data on the impact of membrane perforation on graft stability and implant survival.
